# Vitamin D and health-related quality of life in a community sample of older Canadians

**DOI:** 10.1007/s11136-014-0696-6

**Published:** 2014-04-24

**Authors:** Y. S. Chao, J. P. Ekwaru, A. Ohinmaa, G. Griener, P. J. Veugelers

**Affiliations:** School of Public Health, University of Alberta, 3-50 University Terrace, 8303 – 112 Street, Edmonton, AB T6G 2T4 Canada

**Keywords:** Health-related quality of life, Vitamin D, Public health, Disease prevention

## Abstract

**Purpose:**

To assess how vitamin D status is associated with health-related quality of life (HRQOL) among older residents of Canada.

**Design:**

We analysed baseline data of 1,493 Canadians aged 50 years and over in Alberta on HRQOL (EQ-5D-5L) and serum 25-hydroxyvitamin D (25(OH)D) as a measure of vitamin D status. We applied multivariable regression methods to examine the association between vitamin D status and each of the five dimensions and the summary index of the EQ-5D-5L.

**Results:**

Participants with higher serum 25(OH)D levels were significantly less likely to report problems with mobility, usual activities, and depression and anxiety. Specifically, age- and gender-adjusted odds ratios for reporting problems with mobility, usual activities, and depression and anxiety were 0.58 (95 % confidence interval 0.44–0.78), 0.67 (0.50–0.89), and 0.67 (0.51–0.88) per 100 nmol/L increase in 25(OH)D, respectively. No significant associations were observed for problems with self-care and with pain and discomfort. HRQOL scores combining the responses of each of the five dimensions increased significantly with increasing serum 25(OH)D levels.

**Conclusions:**

This is the first study to reveal the importance of vitamin D for the five dimensions of HRQOL in a community-based sample. The observed associations of vitamin D and HRQOL call for intervention studies to strengthen the evidence of the potential benefits of vitamin D supplementation for HRQOL among older adults.

## Introduction

Vitamin D has been shown to affect bone health and various chronic diseases [1]. To mediate on the burden of some of these conditions, the Institute of Medicine and Health Canada recommend vitamin D supplementation of 600 IU per day for adults and 800 IU per day for those above the age of 70 years [2, 3]. Supplementation may be particularly important to residents of northern geographies, such as Canada, as subcutaneous creation of vitamin D by sun exposure is limited [4]. In Canada, vitamin D deficiency and insufficiency continue to be prevalent despite the recommendations for Vitamin D supplementation [5]. Specifically, the Canadian Health Measures Survey had shown that 4.1 and 10.6 % (95 % confidence interval 2.9–5.8 and 8.2–13.6 %, respectively) of Canadians aged 6–79 years had serum 25-hydroxyvitamin D <27.5 and 37.5 nmol/L, respectively [5]. We recently showed that the rates of deficiency and insufficiency were 3 and 8 %, respectively, in a sample of working age Canadians [6].

For older populations, several vitamin D supplementation studies have suggested, though not consistently, less functional limitation [7], prevention of falls [8–10], reductions in fractures [11–13], and mental health benefits [14, 15]. A meta-analysis by Bischoff-Ferrari et al. [9] concluded that in order to achieve a reduction in falls a supplementation dose of 700–1,000 IU per day is needed which is close to what is currently recommended by the Institute of Medicine. The above studies suggest a relationship of vitamin D and objectively measured health conditions. Health-related quality of life (HRQOL) aims to quantify the subjective experiences of the consequences of these health conditions. The importance of vitamin D for HRQOL has been less studied. Huang et al. [16] attributed improvements in quality of life along with improvements in pain and sleep to vitamin D supplementation in a case series of individuals with chronic pain. However, to the best of our knowledge, no earlier studies have examined the association of vitamin D with the full spectrum of HRQOL in healthy populations. We believe that this is particularly important as the Institute of Medicine recommendations are issued to the general public. We also believe these are particular important to Canadians given the relatively high latitude and consequent reliance on vitamin D from diet and supplements. In the present study, we assess the association of vitamin D status and HRQOL among older residents of Canada.

## Methods

This is a cross-sectional study based on information gathered at baseline visits from volunteer participants before starting a wellness programme by the Pure North S’Energy Foundation (PN). PN, a charitable, not-for-profit organization, provides a lifestyle counselling programme as described in more detail elsewhere [6, 17, 18]. As of August of 2012, PN started the recruitment of older residents of the city of Calgary, Alberta, Canada. PN advertised their programme for seniors through local newsletters and through the distribution of flyers in senior homes and community centres. PN would organize weekly information meetings after which attendees could elect to sign up to enrol the programme. The present study pertains to baseline observations of older residents recruited between August 2012 and April 2013.

At their baseline visit, participants completed a survey had their body height and weight measured and their blood drawn for the assessment of serum 25-hydroxyvitamin D (25(OH)D). The survey included the five-level EQ-5D (EQ-5D-5L) to measure health-related quality of life (HRQOL) [19]. The EQ-5D consists of a five-dimensional descriptive system asking whether participants have (1) no problems; (2) slight problems; (3) moderate problems; (4) severe problems; or (5) extreme conditions or are unable to perform or extreme conditions are fully constrained or restricted, with each of the following: (1) mobility; (2) self-care; (3) usual activities; (4) pain or discomfort; and (5) anxiety or depression [19]. HRQOL scores are based on responses to each of the five dimensions and were derived from the USA value sets [20]. The EQ-5D is an established and validated instrument [19] with the major advantages of being short and easy to complete [21].

The survey further included questions on age, gender, and income. Individuals and couples with an annual income of <$25,000 and of <$41,000, respectively [22], were considered as low income. Body mass index (BMI) was calculated on the basis of the measured heights and weights (weights in kilograms divided by the square of height in metres). Individuals with a BMI of 25 or more are considered overweight, and those with a BMI of 30 or more are considered obese [23].

Serum 25(OH)D is the established proxy for vitamin D status. Some have suggested that individuals with serum 25(OH)D levels below 25 nmol/L should be considered vitamin D deficiency and those with serum 25(OH)D levels of 25 or more and <50 nmol/L be considered vitamin D insufficiency. As recent studies have suggested extra-skeletal benefits, such as reduction in colorectal cancer and cardiovascular disease risk, for individuals with serum levels of 75 nmol/L or more [1, 24, 25], and given the large number of observations with serum levels above 75 nmol/L, we further categorized serum levels into ≥75 and <100, ≥100 and <125, and ≥125 nmol/L.

As descriptive statistics, we included the prevalence of problems in the five HRQOL dimensions by vitamin D category and by age, gender, income, and bodyweight status. We tested for differences in the prevalence of HRQOL problems across vitamin D status categories by the use of univariate logistic regression. We applied univariate and multivariable logistic regression to quantify the association of vitamin D status with problems in each of the five HRQOL domains whereby we had dichotomized the latter into ‘having no problems’ (level 1) and ‘having problems’ (levels 2–5 combined). As HRQOL scores were skewed to the left, with values ranging from 0.129 to 1.0, we log-transformed inverse of the HRQOL scores, i.e. log_10_(1.0001 − HRQOL). These ‘log of inverse HRQOL scores’ increase with poorer HRQOL and constitute the dependent variables in univariate and multivariable linear regression analyses to quantify the significance of vitamin D status for overall HRQOL. *p* values <0.05 were considered statistically significant.

PN anonymized their data prior to forwarding it to the University of Alberta for statistical analysis. The analysis was conducted using STATA version 12 (College Station, Texas). The Human Research Ethics Board of the University of Alberta approved the data access and analysis for this study.

## Results

The mean serum 25(OH)D level among the 1,493 participants was 92.44 nmol/L and ranged from 12 to 319 nmol/L. Thirteen participants (0.9 %) had serum 25(OH)D levels of <25 nmol/L, and 116 (7.8 %) had 25(OH)D levels between 25 and 50 nmol/L (Table 1). Table 1 shows prevalence of problems by vitamin D status for each of the five EQ-5D dimensions and for any of these five problems. Though there are some slight fluctuations, the percentages of participants having problems in each of the five EQ-5D dimensions generally declined with higher categories of serum 25(OH) D. Table 1 also shows associations between age, gender, income, and body weight and HRQOL. These associations are mostly substantial in magnitude and statistically significant.

**Table 1:** Prevalence of problems in the EQ-5D dimensions among 1,493 study participants

Variable		N	Problems with mobility		Problems with self-care		Problems with usual activities		Problems with pain and discomfort		Problems with depression and anxiety		Any problem	
			%	*p* value	%	*p* value	%	*p* value	%	*p* value	%	*p* value	%	*p* value
*25(OH)D level*														
<25 nmol/L		13	53.8	0.65	7.7	0.84	38.5	1.00	76.9	0.58	61.5	0.64	92.3	0.88
≥25, <50 nmol/L		116	47.4	0.98	12.1	0.03	51.7	<.01	80.2	0.48	62.1	0.18	91.4	0.89
≥50, <75 nmol/L		385	47.5	ref.	6.2	ref.	38.4	ref.	83.1	ref.	55.1	ref.	90.9	ref.
≥75, <100 nmol/L		461	39.0	0.01	6.3	0.97	38.4	0.99	79.8	0.23	54.4	0.86	89.2	0.42
≥100, <125 nmol/L		290	35.2	<.01	5.5	0.71	33.8	0.22	79.7	0.26	53.4	0.68	85.9	0.04
>125 nmol/L		228	34.2	<.01	7.5	0.56	35.1	0.41	78.9	0.21	49.1	0.15	86.0	0.06
*Gender*														
Female		971	40.8	0.78	6.7	0.88	39.5	0.10	82.9	<.01	59.5	<.01	90.6	<.01
Male		522	40.0	ref.	6.9	ref.	35.2	ref.	76.1	ref.	44.4	ref.	85.1	ref.
*Age (years)*														
50–59		356	36.2	<.01	5.9	<.01	40.4	0.01	82.3	0.70	65.7	<.01	91.9	0.38
60–69		687	38.9	<.01	5.1	<.01	34.2	<.01	80.5	0.99	53.9	0.16	88.2	0.93
70–79		363	43.0	<.01	8.0	<.01	38.8	<.01	78.8	0.72	45.7	0.97	86.5	0.60
80+		87	60.9	ref.	18.4	ref.	55.2	ref.	80.5	ref.	46.0	ref.	88.5	ref.
*Income*														
Other		853	33.5	ref.	4.1	ref.	30.4	ref.	77.7	ref.	51.2	ref.	86.3	ref.
Low income		640	49.8	<.01	10.3	<.01	48.3	<.01	84.2	<.01	58.3	<.01	91.9	<.01
*Weight status*														
Underweight or normal weight		479	26.9	ref.	5.8	ref.	29.6	ref.	76.0	ref.	54.5	ref.	86.0	ref.
Overweight		593	40.5	<.01	5.2	0.69	36.9	0.01	80.8	0.05	52.6	0.54	89.4	0.08
Obesity		421	56.1	<.01	10.0	0.01	49.2	<.01	85.3	<.01	56.3	0.59	90.7	0.03

Ref.: reference category, 25(OH)D: serum 25-hydroxyvitamin D level, *p* value: Compared with the reference category

Table 2 shows the unadjusted and adjusted associations between 25(OH)D levels and having problems in each of the five EQ-5D dimensions. In the unadjusted analysis, each 100 nmol/L increase in 25(OH)D was significantly associated with a reduction in the odds of having problems with mobility (39 %), usual activities (30 %), pain and discomfort (26 %), and any problem (37 %). These associations remained statistically significant after adjusting for age and gender. Further, adjustment for income and body weight reduced the magnitude of the associations and only the association with anxiety or depression and any problem remained statistically significant.

**Table 2:** Changes in the odds of each EQ-5D dimension problems per 100 nmol increase in 25(OH)D/L among 1,493 study participants

Problem	Unadjusted			Adjusted for age and gender			Adjusted for age, gender, income, and weight status		
	Odds ratio	95 % CI	*p* value	Odds ratio	95 % CI	*p* value	Odds ratio	95 % CI	*p* value
Problems with mobility	0.61	0.46–0.80	<.01	0.58	0.44–0.78	<.01	0.82	0.61–1.10	0.18
Problems with self-care	0.89	0.52–1.53	0.68	0.88	0.51–1.52	0.65	1.11	0.65–1.91	0.70
Problems with usual activities	0.70	0.53–0.92	0.01	0.67	0.50–0.89	<.01	0.87	0.65–1.18	0.37
Problems with pain and discomfort	0.85	0.61–1.17	0.32	0.79	0.57–1.10	0.16	0.95	0.67–1.34	0.77
Problems with depression and anxiety	0.74	0.57–0.97	0.03	0.67	0.51–0.88	<.01	0.71	0.53–0.94	0.02
Any problem	0.63	0.43–0.91	0.02	0.58	0.39–0.84	<.01	0.66	0.44–0.98	0.04

*CI* confidence interval, *25(OH)D* serum 25-hydroxyvitamin D level

**Fig. 1:**
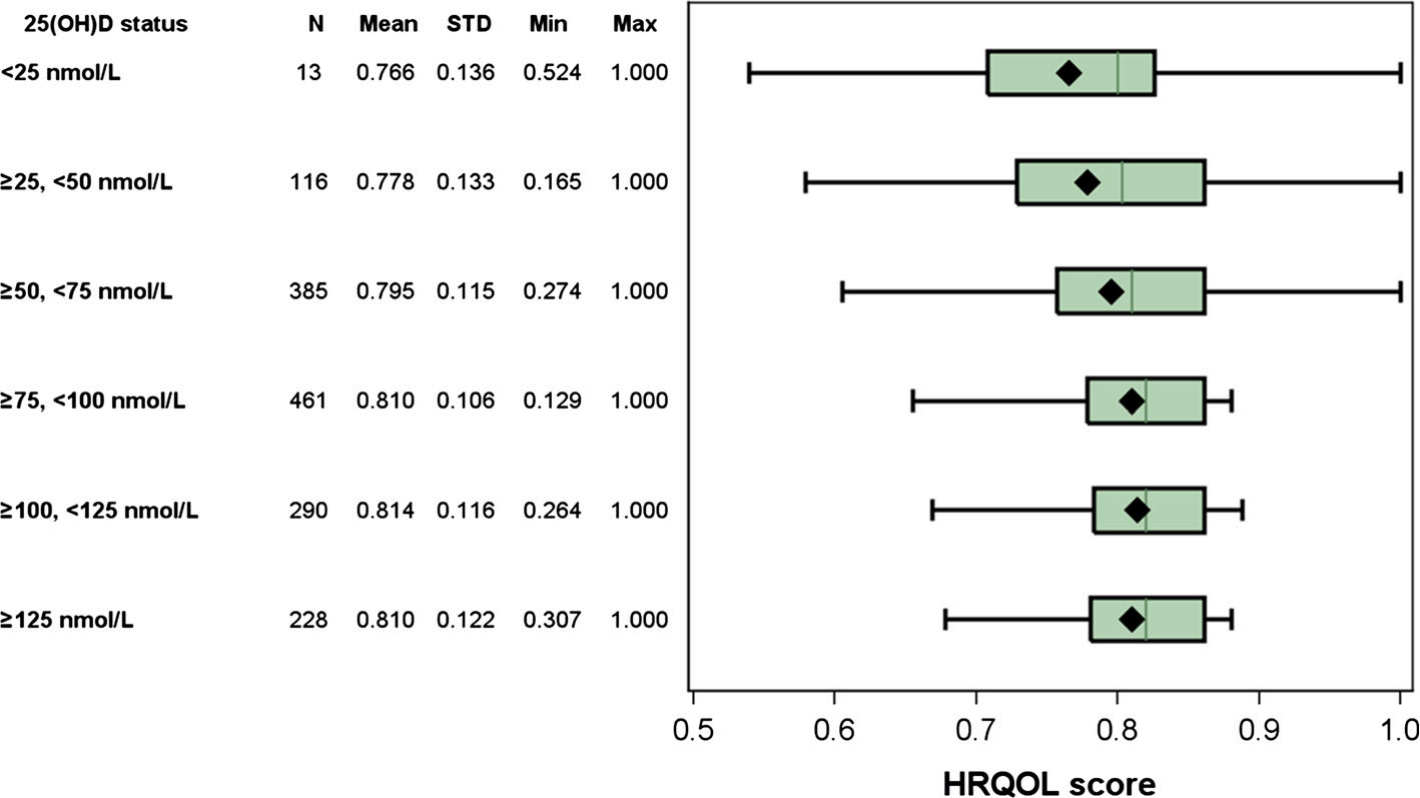
Mean HRQOL score by vitamin D category among 1,493 study participants.* 25(OH)D* serum 25-hydroxyvitamin D level,* HRQOL* health-related quality of life.

Figure 1 shows the mean values of HRQOL scores measured with EQ-5D-5L by vitamin D status. The mean HRQOL scores increased with increasing levels of vitamin D. Table 3 shows unadjusted and adjusted associations between the log of inverse HRQOL score and vitamin D levels, gender, age, weight status, and income. After adjusting for gender, age, weight status, and income, each 100 nmol/L increase in vitamin D level was associated with a 0.15 (95 % CI 0.01, 0.29) statistically significant reduction in the log of inverse HRQOL score, which corresponds to a statistically significant increase in HRQOL score. A reduction in 0.15 in the log of inverse HRQOL score (1 − HRQOL) is equivalent to an increase in HRQOL score of about $29 \left( {\frac{{\text{1} - {\text{HRQOL}}_{0} }} {{{\text{HRQOL}}_{0} }}} \right) \%$ at any given initial HRQOL score ${\text{HRQOL}}_{0}$. Therefore, at a HRQOL score of 0.5 a 100 nmol/L increase in 25(OH)D results in a 29 % increase in HRQOL score. The percentage increase is higher than 29 % if the initial HRQOL score is below less 0.5 and <29 % if the initial HRQOL score is above 0.5.

**Table 3:** Effect of serum 25(OH)D levels, gender, age, weight status, and income on the log-transformed inverse of health-related quality of life score (i.e. log_10_[1.0001 − HRQOL]) among 1,493 study participants

Variable	Unadjusted		Adjusted for age and gender		Adjusted for age, gender, income, and weight status	
	*β* (95 % CI)	*p* value	*β* (95 %CI)	*p* value	*β* (95 % CI)	*p* value
Serum 25(OH)D (100 nmol/L)	−0.19 (−0.32,−0.05)	0.009	−0.22 (−0.36,−0.08)	0.002	−0.15 (−0.29,−0.01)	0.042
*Gender*						
Female	ref.		ref.		ref.	
Male	−0.20 (−0.31,−0.09)	<.001	−0.22 (−0.33,−0.10)	<.001	−0.22 (−0.33,−0.10)	<.001
*Age(years)*						
50–59	ref.		ref.		ref.	
60–69	−0.14 (−0.27,0.00)	0.050	−0.11 (−0.25,0.02)	0.095	−0.15 (−0.28,−0.01)	0.034
70–79	−0.18 (−0.33,−0.02)	0.025	−0.16 (−0.32,−0.01)	0.037	−0.22 (−0.38,−0.07)	0.005
80+	−0.07 (−0.32,0.18)	0.587	−0.04 (−0.29,0.20)	0.732	−0.12 (−0.37,0.12)	0.330
*Weight status*						
Underweight or normal weight	ref.				ref.	
Overweight	0.12 (−0.01,0.24)	0.073			0.16 (0.03,0.28)	0.018
Obesity	0.21 (0.07,0.35)	0.003			0.21 (0.06,0.35)	0.005
*Income*						
Low income	0.24 (0.14,0.35)	<.001			0.23 (0.12,0.34)	<.001
Other	ref.				ref.	

In the above analyses, interaction terms of age and serum 25(OH)D were not associated with health-related quality of life in a statistical significantly manner

*β* coefficient,* CI* confidence interva,* 25(OH)D* serum 25-hydroxyvitamin D leve,* Ref*. reference category,* HRQOL* health-related quality of life

## Discussion

This is the first study to report on the associations of vitamin D with each of the five dimensions of quality of life. The study revealed significant associations of vitamin D with problems with mobility, usual activities, and depression and anxiety. It further revealed that an increase of 100 nmol/L of serum 25(OH)D is associated with an average increase of 29 % in HRQOL.

The average serum 25(OH)D level of participants was 92.44 nmol/L which is substantially higher than the average among Canadians aged 6 to 79 (67.7 nmol/L) and among Canadians 60–79 years of age (72.0 nmol/L) [5]. Self-selection of health aware individuals interested to participate in the wellness programme may have contributed to this. Health aware individuals may eat healthier and comply better with the existing vitamin D supplementation recommendations. This self-selection may particularly apply to individuals with pain and discomfort. We observed that approximately 80 % of participants reported problems with pain and discomfort. This percentage seems higher than those of a population-based survey using EQ-5D-3L conducted in 2010 in the same province where 57 % of participants between the ages of 45 and 64 years and 66 % of participants 65 years of age and older reported problems with pain and discomfort [26]. Subjects experiencing pain and discomfort may be particularly motivated to participant in the programme.

Gender and age are established determinants of vitamin D status [6, 17] and of quality of life [20]. It is, therefore, essential to adjust for the confounding influence of gender and age as we did in this study. We observed that vitamin D was associated with three of the five EQ-5D dimensions: mobility, usual activities, and depression or anxiety. The absence of a statistical significant association with self-care may stem from the very low prevalence of reported problems with self-care in this population. Bias by indication [27], whereby individuals with symptoms, pain, and illness are more likely to comply with recommendations, may have led to an underestimation of the strength of the association with each of the five dimensions. However, this may be particularly true for problems with ‘pain and discomfort’ and explain why no association for this dimension was observed.

Vitamin D has been shown to lead to better muscle strength [28], and, both in observational studies and clinical trials, vitamin D supplementation has been shown to benefit mobility and functional status in terms of prevention of falls [9] and improvement of physical function [29]. Consistent with our observations that individuals with a better vitamin D status reported less problems with mobility and usual activities, these findings [9, 29] seem to suggest less frailty among individuals with a good vitamin D status, though a recent meta-analysis reported that vitamin D supplementation among unselected community-dwelling individuals does not change risk for skeletal or non-skeletal outcomes by more than 15 % [30].

Vitamin D has also been shown to be associated with less depression [14] which seems consistent with our observation that higher vitamin D serum levels are associated with less problems with depression and anxiety. Though the associations with several outcomes are probable, more research is needed for more convincing evidence [31], which may particularly apply to mental health outcomes.

The Institute of Medicine recommendations are issued to the general public, but no earlier study had reported on the association between vitamin D and quality of life in a general population. Though we conducted this in a community sample, caution is warranted when it comes the generalizing our findings as this sample may be self-selected towards a health aware subpopulation with a relatively high prevalence of problems with pain and discomfort. Despite the large sample size, a second study limitation relates to the small number of participants that reported problems with self-care, which hampered some of the analyses. Further, although we did adjust for the confounding effect of age, sex, income, and body weight status, we acknowledge that other factors may have confounded the observed association of 25(OH)D and HRQOL. Further, limitations relate to the cross-sectional design and the existence of reverse causation whereby, for example, individuals with limited mobility due to chronic diseases have less opportunity to get outdoors. A final limitation relates to the use of self-reported information that is prone to error, though the EQ-5D is an established and validated instrument [19] whereby the reporting error is expected to be limited given that it is short and easy to complete [21].
